# Analysis of Chemical Variations between Crude and Salt-Processed *Anemarrhenae rhizoma* Using Ultra-High-Performance Liquid Chromatography–Mass Spectrometry Methods

**DOI:** 10.3390/molecules23010023

**Published:** 2017-12-22

**Authors:** De Ji, Xiaonan Su, Ziyan Huang, Lialin Su, Lin Li, Tulin Lu

**Affiliations:** 1 College of Pharmacy, Nanjing University of Chinese Medicine, Nanjing 210023, China; brmoonsu@163.com (X.S.); huangziyan115@gmail.com (Z.H.); sulianlin1989@163.com (L.S.); ltl209@163.com (T.L.); 2 State Key Laboratory Cultivation Base for TCM Quality and Efficacy, Nanjing University of Chinese Medicine, Nanjing 210023, China; 3 Key Research Laboratory of Chinese Medicine Processing of Jiangsu Province, Nanjing University of Chinese Medicine, Nanjing 210023, China

**Keywords:** *Anemarrhenae rhizoma*, chemical profile, UPLC–QTOF-MS, salt processing, furostanol saponins

## Abstract

The present study was designed to systematically investigate the chemical profile differences between crude *Anemarrhenae rhizoma* (CAR) and salt-processed *Anemarrhenae rhizoma* (SAR). Ultra-high-performance liquid chromatography–quadrupole time-of-flight mass spectrometry (UHPLC–QTOF-MS), coupled with multivariate statistical analysis was used for the discrimination of chemical profiles and the identification of the differentiation of the chemical constitutions of CAR and SAR. In addition, seven main constituents of CAR and SAR were simultaneously determined by ultra-high-performance liquid chromatography–quadrupole mass spectrometry (UHPLC–MS) for analyzing the content variations. A total of 24 components were found to be the main contributors to the significant difference between CAR and SAR. The structures of the marker compounds were identified based on their chromatographic behaviors, intact precursor ions, and characteristic MS fragmentation patterns. The potential structural transformation mechanism of furostanol saponins during salt processing was explored. The results may provide a scientific foundation for deeply elucidating the processing mechanism of *Anemarrhenae rhizoma*.

## 1. Introduction

Chinese medicine processing is a traditional pharmacy technology based on the requirements of clinical medication, the nature of raw drugs, and the different dispensing demands under the guidance of traditional Chinese medicine (TCM) theory [1]. The purposes of processing are to remove impurities, increase potency, reduce toxicity, reduce unpleasant odors, and prepare formulations more easily, etc. [2]. The processed medicinal material products are called decoction pieces (“yinpian” in Chinese), which are clinical prescription drugs in TCM and are the raw materials of Chinese patent medicines [3].

The chemical compounds of TCMs are the base materials for the prevention and treatment of disease [4,5]. Research has demonstrated that the effect of Chinese medicine processing is closely related to process-induced chemical changes. For instance, the chemical composition of steamed and charred pieces of rhubarb is considerably different from its raw materials, as a result of anthraquinone glycosides and stilbene glycosides decomposing into aglycones and glucoses during the process of heating [6]. The contents of quercetin and the total flavonoids were significantly increased after the salt-processing of *Semen cuscutae*, which in turn, increased the sex hormone level, improved immune function as well as improved the antioxidant effect on Kidney-Yang deficiency in rats [7]. However, the majority of mechanisms in herbal medicine processing are still unclear. Consequently, the screening and distinguishing of crude and processed herbs are of benefit to elucidating processing mechanisms and ensuring quality control of processed herbs.

*Anemarrhenae rhizoma* (AR, “zhimu” in Chinese), the dried rhizome of *Anemarrhena asphodeloides* Bge., is a well-known traditional Chinese medicinal herb used for removing heat, quenching fire, promoting the production of body fluids, and relieving dryness syndrome. The processing of AR has a long history and a variety of excipients such as rice wine, salt solution, and wheat bran have been used in it. Among them, salt-processing is the most widely used one, in which AR is stir-heated with a salt solution, as documented in the 2015 edition of the Chinese pharmacopoeia [8]. It is used in many Chinese traditional patent formulations [9]. In recent pharmacological studies, salt-processed AR showed improved effects on clearing asthenic fever, inhibiting α-glucosidase activity, and on antibacterial activity, etc. [10,11]. However, the differences in the chemical compositions between crude *Anemarrhenae rhizoma* (CAR) and salt-processed *Anemarrhenae rhizoma* (SAR) have not been studied.

In this study, in order to conduct a comprehensive comparison of CAR and SAR, an ultra-high-performance liquid chromatography–quadrupole time-of-flight mass spectrometry (UHPLC–QTOF-MS) along with statistical analyses, including principal component analysis (PCA) and the *t*-test methods were developed to investigate the chemical composition differentiation of CAR and SAR. Meanwhile, UHPLC-MS was used to analyze the content variations between CAR and SAR, and seven major compounds were determined. The present study may provide a scientific foundation for deeply elucidating the salt-processing mechanism of AR, thus benefiting future research into their pharmacological activities.

## 2. Results and Discussion

### 2.1. Multivariate Data Analyses and Identification of Characteristic Chemical Compositions Differentiation of CAR and SAR

**Figure 1:**
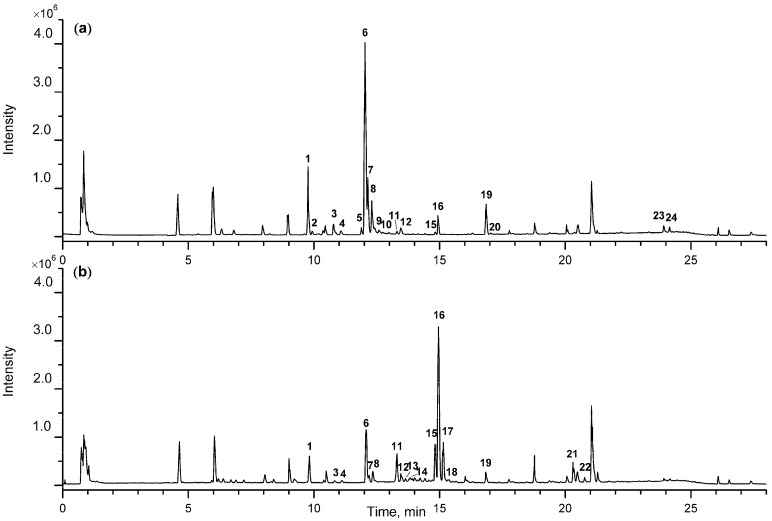
The typical total ion chromatograms (TIC) of crude *Anemarrhenae rhizoma* (CAR) (**a**) and salt-processed *Anemarrhenae rhizoma* (SAR) (**b**) in negative ion mode.

**Figure 2:**
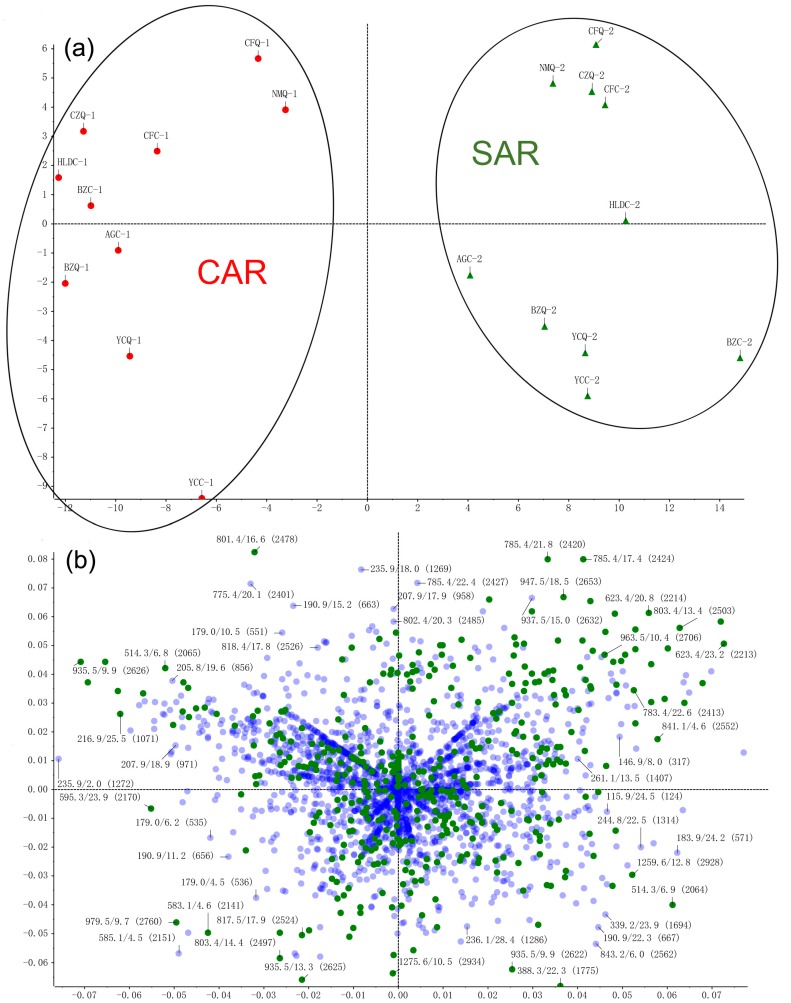
The final principal component analysis (PCA) score plot (**a**) and loading plot (**b**) of CAR and SAR in negative ion mode.

Under the optimized conditions, the samples were scanned in both positive and negative ion modes. It was found that negative ion mode has a higher sensitivity and produces clearer mass spectra than in positive ion mode. However, positive ion mode produces more fragments, which is beneficial for the structural identification of aglycone-type steroidal saponins in AR. Typical total ion chromatograms (TICs) for CAR and SAR, both in negative ion mode are shown in Figure 1. The final PCA score plot and loading plot in negative ion mode are shown in Figure 2. As displayed in the score plot, 20 samples were divided into two groups, and the division corresponded with their specifications as CAR and SAR, indicating that certain differences exist in the chemical profiles of the two groups. In order to investigate the key markers that contribute most to the difference between CAR from SAR, the *t*-test was performed. The results demonstrate that 24 peaks are the main contributors to the significant differences between CAR and SAR (*p* < 0.05) (Table 1). The results of both the PCA and the *t*-test illustrate that chemical profile changes occur in AR upon processing.

**Figure 3:**
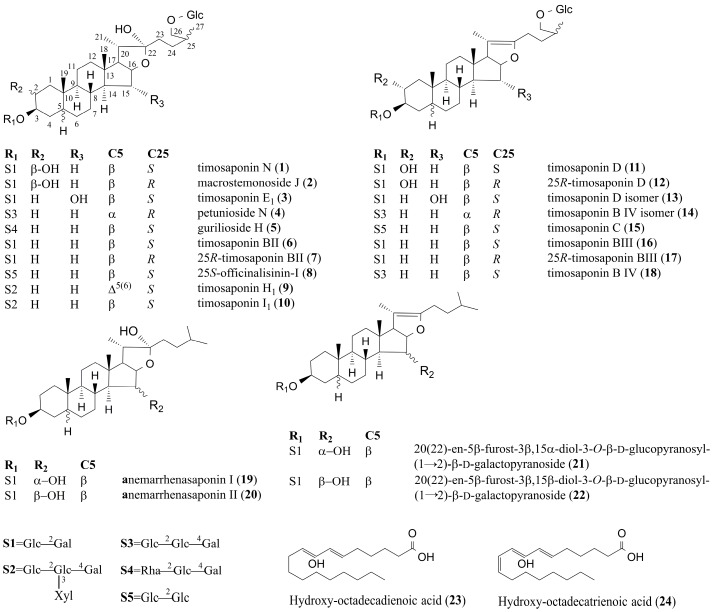
The structures of 24 identified compounds.

After processing the data by multivariate statistical analysis, the marker components were listed for further verification. Among the 24 key characteristic compounds, timosaponin N (peak 1), timosaponin E_1_ (peak 3), timosaponin BII (peak 6), timosaponin BIII (peak 16), and anemarrhenasaponin I (peak 19) were unambiguously identified by comparison with reference compounds. For the unknown compounds, we first analyzed the molecular ions and derived accurate molecular formulae for possible candidate structures using PeakView. The identities of the peaks were then surmised by the fragmentation patterns, chromatographic behaviors, and by comparing them with the chemical components database of AR. The structures of all the identified compounds are shown in Figure 3.

**Figure 4:**
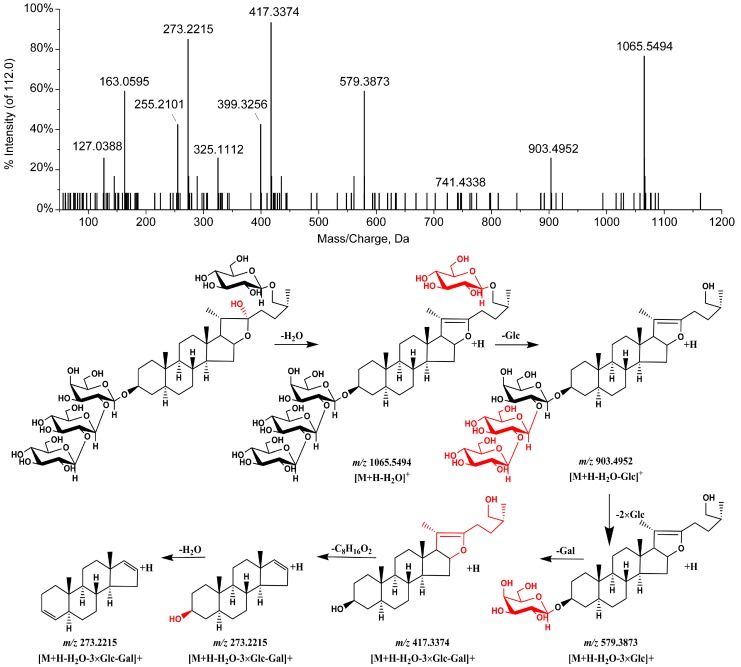
MS^2^ spectrum and proposed fragmentation pathways of peak 4 in the positive ion mode.

Based on the above methods, peak 2 is found to have an identical molecular formula and fragmentation pattern to those of timosaponin N and is tentatively identified as macrostemonoside J based on the retention time and literature reports [12]. Peaks 4 and 5 show similar fragmentation patterns to those of timosaponin BII. Peak 4 presents fragments at *m*/*z* 1065.5494 [M + H − H_2_O]^+^, 903.4952 [M + H − H_2_O − 162]^+^, 579.3873 [M + H − H_2_O − 162 − (3 × 162)]^+^, 417.3374 [M + H − H_2_O – 162 − (4 × 162)]^+^, 273.2205 [417.3400 − C_8_H_16_O_2_]^+^, and 255.2101 [273.2214 − H_2_O]^+^ in ESI^+^ mode, and peak 5 presents fragments at *m*/*z* 1049.5289 [M + H − H_2_O]^+^, 903.4882 [M + H − H_2_O − 146]^+^, 741.3754 [M + H − H_2_O – 146 − 162]^+^, 579.3326 [M + H − H_2_O − 146 − (2 × 162)]^+^, 417.2795 [M + H− H_2_O − 146− (3 × 162)]^+^, 273.1602 [417.3400 − C_8_H_16_O_2_]^+^, and 255.1455 [273.2214 − H_2_O]^+^ in ESI^+^ mode, indicating that the compounds responsible for peaks 4 and 5 have a hexosyl residue and a deoxyhexosyl residue, respectively, more so than for timosaponin BII. Thus, peaks 4 and 5 are tentatively identified as petunioside N and curilioside H [13,14]. The ion fragmentation spectra from the tandem mass spectrometry (MS/MS) analysis and the proposed fragmentation pattern of peak 4 are shown in Figure 4. Peaks 7 and 8 have the same precursor ions (*m*/*z* 903.4931 [M + H − H_2_O]^+^, and 903.4940 [M + H − H_2_O]^+^) as timosaponin BII, and are identified as isomers of timosaponin BII. Based on their retention times, and by comparison with known compounds in the literature [12,15], peaks 7 and 8 are identified as 25*R*-timosaponin BII and 25*S*-officinalisinin-I. Peaks 9 and 10 present precursor ions at *m*/*z* 1195.4256 [M + H − H_2_O]^+^ and 1197.4558 [M + H − H_2_O]^+^, respectively, and are identified as timosaponin H_1_ and timosaponin I_1_ based on the fragmentation, retention time, and by comparison with known compounds in the literature [12,16].

Peaks 11–15, 17, and 18 all have aglycone skeletons similar to that of timosaponin BIII. The [M + H]^+^ precursor ions for peaks 11–15, 17, and 18 are observed at *m*/*z* 919.4893, 919.4901, 919.4889, 1065.5008, 903.4984, 903.4958, and 1065.5078, respectively. Based on their fragment ions and by comparison with the known compounds in AR [12,14,17], peaks 11–15, 17, and 18 are identified as timosaponin D, 25*R*-timosaponin D, (25*S*)-26-*O*-β-d-glucopyranosyl-5β-furostane-20(22)-en-3β,15*α*, 26-triol-3-*O*-β-d-glucopyranosyl-(1→2)-β-d-galactopyranoside, (25*R*)-26-*O*-β-d-glucopyranosyl-5*α*-furostane-20(22)-en-3β, 26-diol-3-*O*-β-d-glucopyranosyl-(1→2)-β-d-glucopyranosyl-(1→4)-β-d-galactopyranoside, timosaponin C, 25*R*-timosaponin BIII, and timosaponin B IV, respectively. 

Peak 20 had the same formula and MS data as peak 19. It was reported that the retention times of steroidal saponins with the 15α-OH configuration were shorter than those with the 15β-OH configuration on a C_18_ column [12]. Based on the retention time and by comparison with a known compound [18], peak 20 is tentatively identified as anemarrhenasaponin II, an isomer of anemarrhenasaponin I. Peaks 21 and 22 produce precursor ions at *m*/*z* 785.4321 [M + HCOO]^−^ and 785.4323 [M + HCOO]^−^ in ESI^−^ mode, and 741.4308 [M + H]^+^ and 741.4303 [M + H]^+^ in ESI^+^ mode, respectively, which were 16 Da less than anemarrhenasaponin I and anemarrhenasaponin II. [M + H]^+^ ions were observed, which means that there was no hydroxyl group at the positions of C-22 for peaks 21 and 22. Besides, peaks 21 and 22 showed the same fragmentation behaviors as anemarrhenasaponin I and anemarrhenasaponin II. Thus, peaks 20 and 21 are tentatively identified as 20(22)-en-5β-furost-3β,15α-diol-3-*O*-β-d-glucopyranosyl-(1→2)-β-d-galactopyranoside and 20(22)-en-5β-furost-3β,15β-diol-3-*O*-β-d-glucopyranosyl-(1→2)-β-d-galactopyranoside, respectively, which are two newly generated compounds. 

Peaks 23 and 24 are tentatively identified as hydroxy-octadecadienoic acid and hydroxy-octadecatrienoic acid, respectively, by comparing the *m*/*z* of the quasi-molecular ion of each compound in ESI^−^ spectra with those in the literature [19], and in the relevant databases (such as ChemSpider, HMDB, PubChem, and MassBank).

### 2.2. Determination of the Seven Main Components in CAR and SAR

Timosaponin N, timosaponin E_1_, timosaponin BII, timosaponin BIII, anemarrhenasaponin I, timosaponin AII, and timosaponin AIII were quantified by UHPLC–MS. All seven analytes showed good linearity (*r*^2^ > 0.9997) within the test ranges. The limits of detection (LODs) of the seven compounds are estimated to be 1.0–4.8 ng/mL, and the limits of quantification (LOQs) are 3.5–19.0 ng/mL. These data show that the selected ion mode (SIM) is sensitive enough to determine the analytes. The values of these seven compounds are listed in Table 2. The relative standard deviation (RSD) values for precision of all these seven compounds are in the range 0.17–1.26% for the intraday assays and 0.85–3.26% for the interday assays. The repeatability RSD is less than 2.94%. The RSD values for the storage stability are less than 2.11%. The recoveries of the method are in the range of 96.35–103.01% with RSDs lower than 2.90%, indicating that the method is accurate for determining the seven compounds. All of these values indicated that the system was suitable for the quantitative analysis.

The validated UHPLC–MS method was used to analyze 20 batches of samples, including 10 batches of CAR and 10 batches of SAR. The analytes were quantified based on their respective calibration curves. The quantitative results are presented in Table 3. It is clear from the results that the timosaponin N, timosaponin E_1_, timosaponin BII, and the anemarrhenasaponin I contents decrease significantly upon processing (*p* < 0.01 or 0.05), while the timosaponin BIII content increases markedly (*p* < 0.01). Timosaponin AII and timosaponin AIII contents showed no obvious change.

### 2.3. Compounds Changed upon Processing

**Figure 5:**
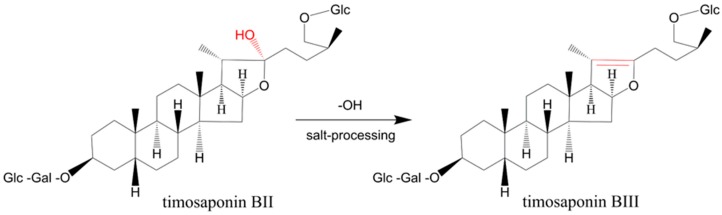
Transformation of timosaponin II during salt-processing of *Anemarrhenae rhizoma* (AR).

According to the results, the chemical profiles of the CAR samples are quite different to those of the SAR samples and 24 peaks were found to show significant differences between CAR and SAR. The intensities of 14 peaks among 24 peaks, including those for 12 furostanol saponins, timosaponin N, macrostemonoside J, timosaponin E_1_, petunioside N, curilioside H, timosaponin BII, 25R-timosaponin BII, 25S-officinalisinin-I, timosaponin H1, timosaponin I1, anemarrhenasaponin I, and anemarrhenasaponin II, are higher in CAR than those in SAR. These compounds are all furostanol saponins sharing a common feature that they all contain a C-22–OH moiety. Conversely, the intensities of the other ten peaks in CAR are lower than those in SAR. These compounds are all furostanol saponins containing a C-20–C-22 double bond. Therefore, it can be concluded that the furostanol saponins in AR that contain a C-22–OH moiety may undergo a C-22–O bond cleavage during salt-processing. The transformation of timosaponin II was illustrated as an example (Figure 5). This result is in accordance with reports in the literature that the C-22 position of steroidal saponins is an active site and that the C22–OH group may be easily lost or substituted [20,21]. In addition, the quantitative result shows that there was no obvious variation in the content of spirostanol saponins such as timosaponin AII and timosaponin AIII. Moreover, the intensities of the peaks for hydroxy-octadecadienoic acid and hydroxy-octadecatrienoic acid clearly decrease, which may be attributed to the heating process.

## 3. Materials and Methods

### 3.1. Materials and Reagents

Purified water was obtained from a Milli-Q water purification system (Millipore Corporation, Bedford, MA, USA). Acetonitrile (ACN, LC-MS grade) was purchased from E. Merck (Merck, Darmstadt, Germany). Formic acid with a purity of 99% (UHPLC grade) was purchased from Anaqua Chemical Supply (ACS, Houston, TX, USA). HPLC grade ethanol (Nanjing Chemical Reagent Factory, Nanjing, China) was used for sample preparation. Authentic standards of timosaponin BII were obtained from the National Institute for the Control of Pharmaceutical and Biological Products (Beijing, China). Timosaponin E_1_, timosaponin BIII, anemarrhenasaponin I, timosaponin AIII, and timosaponin AII were purchased from Chengdu Must Bio-technology Co., Ltd. (Chengdu, China). The purities of these compounds were confirmed to be more than 98%. Timosaponin N was isolated in our laboratory, and its structure was confirmed by MS and NMR. Ten batches of AR were collected from wild or cultivated sources in China (Table 4). The voucher specimens were identified by Professor Tulin Lu of the Nanjing University of Chinese Medicine, and deposited at the College of Pharmacy therein. CAR samples were obtained by first removing any foreign matter and washing. They were then softened thoroughly, cut into thin slices, and dried. To obtain SAR, the CAR was thoroughly mixed with salt water (20%, *w*/*w*), fried over a gentle heat until totally dry, and allowed to cool. A ratio of 2 kg of salt for each 100 kg of the crude drug was used [6].

### 3.2. Preparation of Standard Solutions

Reference standards were accurately weighed and then dissolved in ACN/water (70:30, *v*/*v*) to yield 2 mg/mL standard stock solutions. The stock solutions were mixed and diluted to a concentration of 100 μg/mL for injection into the UHPLC–QTOF-MS system. Calibration solutions were prepared by diluting the stock solutions to the required concentrations. The solutions were prepared at six concentrations levels. The concentration ranges were from 2.0 to 16.0 μg/mL for timosaponin N, anemarrhenasaponin I, and timosaponin AII, 0.1 to 6.0 μg/mL for timosaponin E_1_, 20.0 to 120.0 μg/mL for timosaponin BII, and 3.0 to 60.0 μg/mL for timosaponin BIII and timosaponin AIII. All these solutions were stored at 4 °C prior to analysis.

### 3.3. Sample Preparation

The samples were powdered to homogeneity and passed through a 40 mesh sieve. Powdered samples (50 mg) were accurately weighed and extracted with 50 mL 70% ethanol (*v*/*v*) under ultrasonication for 30 min. The extraction solutions were transferred into 50 mL volumetric flasks, which were made up to the mark with the same solvent. The mixtures were centrifuged at 13,000 rpm for 5 min, and the supernatant liquor was used for quantification analysis. The solutions were diluted 10-fold with 70% ethanol for UHPLC–QTOF-MS analysis.

### 3.4. UHPLC–QTOF-MS Analysis

#### 3.4.1. Instrument and Chromatographic Conditions

UHPLC separation was performed on a Shimadzu 30A UHPLC system (Shimadzu, Japan). The separation was carried out on an Agilent Eclipse Plus-C_18_ column (2.1 × 100 mm, 1.8 μm), preceded with an Agilent Eclipse Plus-C_18_ guard column (2.0 × 5 mm, 1.8 μm). The mobile phase consisted of solvent A (0.1% formic acid in water, *v*/*v*) and solvent B (ACN). The optimized UHPLC elution program was as follows: 0–5 min, 5–18% B; 5–10 min, 18–25% B; 10–20 min, 25–50% B; 20–25 min, 50–100% B; 25–28 min, 100% B. The column temperature was maintained at 30 °C. The flow rate was 0.3 mL/min and the injection volume was 2 μL. 

MS detection was performed on a Triple TOF 5600^+^ (AB Sciex, Los Angeles, CA, USA) hybrid triple Q-TOF mass spectrometer, equipped with an electron spray ionization (ESI) source. The MS was operated in both positive and negative ion modes. The operating parameters of the MS analysis were as follows: Ion spray voltages were set at 4500 and 5500 V in the negative and positive ion modes, respectively; turbo spray temperature: 550 °C; declustering potential: 60 V; collision energy: 35 ± 15 eV; nebulizer gas (gas 1): 55 psi; heater gas (gas 2): 55 psi; curtain gas: 35 psi. Nitrogen was used as the nebulizer and auxiliary gas. TOF MS and TOF MS/MS were conducted over *m*/*z* ranges of 100–2000 and 50–1000, respectively. The experiments were run with accumulation times of 200 ms and 80 ms for TOF MS and TOF MS/MS, respectively. Recalibration was carried out at 3 h intervals. In addition, dynamic background subtraction and information dependent acquisition techniques were used to reduce the impact of matrix interference and increase the efficiency of the analyses. All of the operations and acquisitions were controlled using Analyst TF 1.6 software (AB Sciex, Los Angeles, CA, USA). Peakview 1.2 software (AB Sciex, Los Angeles, CA, USA) was used to identify chemical compounds, and Markerview 1.2.1 software (AB Sciex, Los Angeles, CA, USA) was used for the multiple statistical analyses.

#### 3.4.2. Data Analysis

The raw data were collected by Analyst TF 1.6 software. The main parameters used for data gathering were retention time (RT) within the range of 0.5–28 min, mass within the range of 100–2000 Da, and a mass tolerance of 5 ppm. In order to select the chemical markers that differentiate CAR from SAR, the chromatographic peaks in the different chromatograms were considered and an alignment algorithm was performed. The following parameters were used to extract and identify the peaks from the raw data: Minimum retention time: 0.50 min (to discount the void volume); maximum retention time: 28 min (the final time of the chromatographic run); subtraction offset: 10 scans; subtraction multiplication factor: 1.3; noise threshold: 100; minimum spectral peak width: 10 ppm; minimum retention time peak width: 5 scans; retention time tolerance: 0.40 min; mass tolerance: 10 ppm; maximum number of peaks: 5000. These settings allowed the program to find small and narrow mass peaks to be merged during alignment. All of the data were visualized using PCA by Markerview 1.2.1 software (AB Sciex, Los Angeles, CA, USA) to check for outliers and variation trends. Then, the *t*-test was performed to derive a list of peaks that were finally defined as the main contributors to the significant difference between CAR and SAR (*p* < 0.05). PeakView 1.2 software (AB Sciex, Los Angeles, CA, USA) was used for qualitative analyses of the marker compounds, both the Extract Ions Chromatogram (XIC) and the MS Library were applied for the identification of target compounds. In addition, Enhance Peak Find (AB Sciex, Los Angeles, CA, USA), IDA Explorer (AB Sciex, Los Angeles, CA, USA), and Formula Finder (AB Sciex, Los Angeles, CA, USA) were applied for the identification of non-target compounds.

### 3.5. Quantification of Seven Compounds by UHPLC-MS

Chromatographic analysis was performed on an Agilent 1200 system (Agilent, Germany). Separation was achieved using an Agilent SB-C_18_ microbore column (2.1 × 50 mm, 1.8 μm). The column temperature was maintained at 30 °C. The mobile phase consisted of solvent A (0.1% formic acid in water, *v*/*v*) and solvent B (ACN) by a gradient elution of 5% B at 0–0.5 min, 5–20% B at 0.5–1.0 min, 20–22% B at 1.0–3.0 min, 22% B at 3.0–3.5 min, 22–35% B at 3.5–5.0 min, 35–45% B at 5.0–6.0 min, and 45–90% B at 6.0–12.0 min. The flow rate was 0.4 mL/min. The injection volume was 10 μL. Mass spectrometric detection was carried out by a single quadrupole mass spectrometer (Product No. G2710BA, Agilent Corp, Palo Alto, CA, USA) equipped with an Electrospray Ionization (ESI) source. Analysis was carried out in selected ion mode (SIM) at *m*/*z* 935.4, 935.4, 919.6, 901.5, 757.4, 755.4, and 739.5 for timosaponin N, timosaponin E_1_, timosaponin BII, timosaponin BIII, anemarrhenasaponin I, timosaponin AII, and timosaponin AIII, respectively, by Chemstation software (Agilent Technologies, Palo Alto, CA, USA). Other parameters such as drying gas flow, drying gas temperature, capillary temperature, nebulizing gas pressure, and capillary voltage were also optimized to improve the response of all compounds. 

The calibration curves, LODs and LOQs, precision, repeatability, stability, and recovery tests of all seven analytes were determined by the previously mentioned chromatography. The calibration curves were prepared by plotting the peak areas of the target analyte, versus their corresponding concentrations using a least-squares linear regression analysis. The LOD and the LOQ for each analyte were defined as 3- and 10-times the signal-to-noise ratio (S/N), respectively. Intra- and interday variations were assessed to determine the precision of the developed assay. For intraday variability, the samples were analyzed six times on the same day, while for interday variability, the samples were analyzed in triplicate over three consecutive days. The relative standard deviations (RSDs) were calculated as a measure of precision. The method reproducibility was evaluated by six individual preparations of the same sample, and the percentage RSD of the area was calculated. To confirm the stability of the method, the same sample was stored at room temperature and analyzed by replicate injection at 0, 2, 4, 6, 8, 12, and 24 h. Again, the RSD was used to evaluate the method stability. The accuracy of the method was evaluated in triplicate by adding known amounts of the eight standards into the samples at three different levels (50, 100, and 150%) with respect to their corresponding quantities. The percentage recoveries were calculated from the slope and Y-intercept of the calibration curve.

## 4. Conclusions

In the present study, an integrated strategy based on chemical profiling by the use of UHPLC–QTOF-MS, combined with multivariate statistical analysis was established for chemical profile discrimination and chemical marker identification of CAR and SAR. PCA analysis demonstrated that CAR and SAR samples can be easily discriminated, and 24 compounds with changed structures or contents were found and identified by *t*-test analysis, combined with non-target compound analysis in PeakView. Moreover, the simultaneous quantification of seven major compounds was carried out with UHPLC–MS. The established methodology displayed acceptable levels of linearity, precision, repeatability, and accuracy. The qualitative and quantitative results indicated that the contents of furostanol saponins containing C-22–OH moiety had decreased, while the contents of furostanol saponins containing C-20–C-22 double bond had increased after salt-processing. The fatty acid contents had also decreased upon processing. This is the first report on the exploration of rational chemical compositions for the differentiation of CAR and SAR. This analytical strategy is expected to provide new insights for evaluating the quality of processed herbal materials.

## Figures and Tables

**Table 1:** Identification of 24 peaks showing significant difference.

Peak	t_R_	Formula	Negative Ion Mode of ESI-MS (*m*/*z*)			Positive Ion Mode of ESI-MS (*m*/*z*)			Identification	*t*-Value	*p*-Value	Changing Direction ^c^
			Precursor Ion	Selective Ion	MS^2^ Fragmentation	Precursor Ion	Selective Ion	MS^2^ Fragmentation				
1	9.81	C_45_H_76_O_20_	981.4892	[M + HCOO]^−^	935, 773, 611	919.4911	[M − H_2_O + H]^+^	757, 595, 433, 415, 289, 271, 253	Timosaponin N ^a^	2.77	0.01271	↓ *
2	9.97	C_45_H_76_O_20_	981.4925	[M + HCOO]^−^	935, 773, 611	919.4916	[M − H_2_O + H]^+^	757, 595, 433, 415, 289, 271, 253	Macrostemonoside J [12]	2.06	0.03379	↓ *
3	10.78	C_45_H_76_O_20_	981.4914	[M + HCOO]^−^	935, 773, 611	919.4918	[M − H_2_O + H]^+^	757, 595, 433, 415, 289, 271, 253	Timosaponin E_1_^a^	3.25	0.00447	↓ **
4	11.08	C_51_H_86_O_24_	1127.5433	[M + HCOO]^−^	1081, 919, 757, 595	1065.5482	[M − H_2_O + H]^+^	903, 741, 579, 417, 399, 273, 255	Petunioside N [13]	2.21	0.03999	↓ *
5	11.89	C_51_H_86_O_23_	1111.5528	[M + HCOO]^−^	1065, 919, 757, 595	1049.5289	[M − H_2_O + H]^+^	903, 741, 579, 417, 399, 273, 255	Curilioside H [14]	2.92	0.00921	↓ **
6	12.03	C_45_H_76_O_19_	965.5029	[M + HCOO]^−^	919, 757, 595	903.4982	[M − H_2_O + H]^+^	741, 579, 417, 399, 273, 255	Timosaponin BII ^a^	4.10	0.00067	↓ **
7	12.14	C_45_H_76_O_19_	965.4945	[M + HCOO]^−^	919, 757, 595	903.4931	[M − H_2_O + H]^+^	741, 579, 417, 399, 273, 255	25*R*-timosaponin BII [12,15]	4.17	0.00058	↓ **
8	12.30	C_45_H_76_O_19_	965.4943	[M + HCOO]^−^	919, 757, 595	903.4940	[M − H_2_O + H]^+^	741, 579, 417, 399, 273, 255	25*S*-officinalisinin-I [12,15]	2.48	0.03575	↓ *
9	12.60	C_56_H_92_O_28_	1257.5733	[M + HCOO]^−^	1211, 1079, 1049, 917, 755, 593	1195.4256	[M − H_2_O + H]^+^	1033, 901, 739, 577, 433, 415, 271, 253	Timosaponin H_1_ [12,16]	2.73	0.01360	↓ *
10	12.75	C_56_H_94_O_28_	1259.5853	[M + HCOO]^−^	1213, 1081, 919, 757, 595	1197.4558	[M − H_2_O + H]^+^	1065, 903, 741, 579, 435, 417, 273, 255	Timosaponin I_1_ [12,16]	2,17	0.04392	↓ *
11	13.30	C_45_H_74_O_19_	963.4798	[M + HCOO]^−^	917, 755, 593	919.4893	[M + H]^+^	757, 595, 433, 415, 289, 271, 253	Timosaponin D [12,17]	−5.82	1.6189 × 10^−5^	↑ **
12	13.46	C_45_H_74_O_19_	963.4806	[M + HCOO]^−^	917, 755, 593	919.4901	[M + H]^+^	757, 595, 433, 415, 289, 271, 253	25*R*-timosaponin D [12]	3.53	0.00241	↑ **
13	13.64	C_45_H_74_O_19_	963.4793	[M + HCOO]^−^	917, 755, 593	919.4889	[M + H]^+^	757, 595, 433, 415, 289, 271, 253	Timosaponin D isomer [12]	−2.02	0.03806	↑ *
14	13.83	C_51_H_84_O_23_	1109.5370	[M + HCOO]^−^	1063, 901, 739	1065.5008	[M + H]^+^	903, 741, 579, 417, 273, 255	(25*R*)-26-*O*-β-d-glucopyranosyl-5*α*-furostane-20(22)-en-3β, 26-diol-3-*O*-β-d-glucopyranosyl-(1→2)-β-d-glucopyranosyl-(1→4)-β-d-galactopyranoside [12,14]	−2.82	0.01144	↑ *
15	14.82	C_45_H_74_O_18_	947.4865	[M+HCOO]^−^	901, 739, 721, 577	903.4984	[M + H]^+^	741, 579, 417, 273, 255	Timosaponin C [12,14]	−5.24	5.4915 × 10^−5^	↑ **
16	14.95	C_45_H_74_O_18_	947.4851	[M + HCOO]^−^	901, 739, 577	903.4941	[M + H]^+^	741, 579, 417, 273, 255	Timosaponin BIII ^a^	−9.15	3.4202 × 10^−8^	↑ **
17	15.14	C_45_H_74_O_18_	947.4854	[M + HCOO]^−^	901, 739, 577	903.4958	[M + H]^+^	741, 579, 417, 273, 255	25*R*-timosaponin BIII [12,14]	−11.53	9.5454 ×10^−10^	↑ **
18	15.33	C_51_H_84_O_23_	1109.5577	[M + HCOO]^−^	1063, 901, 739	1065.5078	[M + H]^+^	903, 741, 579, 417, 273, 255	Timosaponin B IV [12,14]	−4.44	0.00032	↑ **
19	16.85	C_39_H_66_O_14_	803.4412	[M + HCOO]^−^	757, 595, 433	741.4397	[M − H_2_O + H]^+^	579, 417, 399, 289, 271, 253	Anemarrhenasaponin I ^a^	3.23	0.00464	↓ **
20	17.03	C_39_H_66_O_14_	803.4415	[M + HCOO]^−^	757, 595, 433	741.4398	[M − H_2_O + H]^+^	579, 417, 399, 289, 271, 253	Anemarrhenasaponin II [18]	2.12	0.04854	↓ *
21	20.32	C_39_H_64_O_13_	785.4321	[M + HCOO]^−^	739, 577	741.4308	[M + H]^+^	579, 417, 399, 289, 271, 253	20(22)-en-5β-furost-3β,15α-diol-3-*O*-β-d-glucopyranosyl-(1→2)-β-d-galactopyranoside ^b^	−4.71	0.00017	↑ **
22	20.77	C_39_H_64_O_13_	785.4323	[M + HCOO]^−^	739, 577	741.4303	[M + H]^+^	579, 417, 399, 289, 271, 253	20(22)-en-5β-furost-3β,15β-diol-3-*O*-β-d-glucopyranosyl-(1→2)-β-d-galactopyranoside ^b^	−2.12	0.04033	↑ *
23	23.92	C_18_H_32_O_3_	295.2280	[M − H]^−^	277, 195, 171				Hydroxy-octadecadienoic acid [19]	4.33	0.00040	↓ **
24	24.15	C_18_H_30_O_3_	293.2124	[M − H]^−^					Hydroxy-octadecatrienoic acid [19]	2.39	0.02800	↓ *

Note: ^a^ indicated that the compounds were identified by comparison with reference compounds. ^b^ indicated that the compounds were newly generated compounds. ^c^ * *p* < 0.05, ** *p* < 0.01, compared to CAR.

**Table 2:** Regression equations, linear range, correlation coefficients, limits of detection (LODs), and limits of quantification (LOQs) of eight compounds.

Compounds	Linear Regression	*r*^2^	Linear Range (μg/mL)	LOD (ng/mL)	LOQ (ng/mL)
Timosaponin N	*y* = 68,743*x* + 27,585	0.9997	2.0–16.0	2.8	9.8
Timosaponin E_1_	*y* = 100,376*x* + 13,353	0.9997	0.1–6.0	3.6	11.3
Timosaponin BII	*y* = 13,964*x* + 8444	0.9999	20.0–120.0	4.8	19.0
Timosaponin BIII	*y* = 53,259*x* + 21,310	0.9998	3.0–60.0	2.7	8.6
Anemarrhenasaponin I	*y* = 71,064*x* + 29,916	0.9999	2.0–16.0	3.0	10.0
Timosaponin AII	*y* = 58,520*x* + 17,831	0.9998	2.0–16.0	2.0	7.4
Timosaponin AIII	*y* = 38,170*x* + 32,791	0.9998	3.0–60.0	1.0	3.5

**Table 3:** The contents of seven compounds in ten batches of CAR and SAR (mg/g, mean ± SD, *n* = 3).

No.	Timosaponin N		Timosaponin E_1_		Timosaponin BII		Timosaponin BIII		Anemarrhenasaponin I		Timosaponin AII		Timosaponin AIII	
	CAR	SAR	CAR	SAR	CAR	SAR	CAR	SAR	CAR	SAR	CAR	SAR	CAR	SAR
1	11.03 ± 0.28	5.20 ± 0.13 **	12.57 ± 0.03	7.45 ± 0.02 **	81.58 ± 1.38	72.44 ± 0.61 **	1.44 ± 0.18	11.18 ± 0.57 **	9.16 ± 0.23	7.92 ± 0.10 **	9.09 ± 0.23	9.00 ± 0.23	37.82 ± 0.96	38.33 ± 0.97
2	13.44 ± 0.34	6.82 ± 0.17 **	15.78 ± 0.04	10.79 ± 0.03 *	83.13 ± 1.41	72.80 ± 0.62 **	2.02 ± 0.26	10.53 ± 0.53 **	7.54 ± 0.19	6.07 ± 0.08 *	5.63 ± 0.14	5.41 ± 0.14	26.77 ± 0.68	25.79 ± 0.65
3	12.59 ± 0.32	5.26 ± 0.13 **	19.70 ± 0.05	6.64 ± 0.02 **	91.21 ± 1.54	63.05 ± 0.53 **	1.73 ± 0.22	13.04 ± 0.66 **	7.15 ± 0.18	3.95 ± 0.05 **	2.91 ± 0.07	4.06 ± 0.10	19.94 ± 0.38	21.88 ± 0.56
4	14.47 ± 0.37	5.00 ± 0.13 **	15.98 ± 0.04	8.89 ± 0.02 **	84.31 ± 1.43	71.33 ± 0.60 **	0.63 ± 0.18	12.01 ± 0.61 **	6.54 ± 0.17	5.86 ± 0.07 *	2.26 ± 0.06	2.34 ± 0.06	13.49 ± 0.34	13.46 ± 0.34
5	11.53 ± 0.29	5.65 ± 0.14 **	6.53 ± 0.02	4.07 ± 0.01 *	82.76 ± 1.40	72.01 ± 0.61 **	6.39 ± 0.15	11.58 ± 0.59 **	8.24 ± 0.21	7.30 ± 0.09 *	4.23 ± 0.11	4.41 ± 0.11	35.56 ± 0.90	36.99 ± 0.94
6	9.21 ± 0.23	4.11 ± 0.10 **	19.37 ± 0.05	6.07 ± 0.02 **	91.01 ± 1.54	60.15 ± 0.51 **	1.30 ± 0.17	11.92 ± 0.61 **	9.78 ± 0.25	5.98 ± 0.08 **	6.15 ± 0.16	7.04 ± 0.18	32.63 ± 0.65	32.05 ± 0.81
7	12.58 ± 0.35	5.55 ± 0.14 **	8.47 ± 0.02	4.65 ± 0.01 **	71.68 ± 1.21	55.24 ± 0.47 **	3.32 ± 0.42	5.94 ± 0.81 **	7.12 ± 0.18	5.51 ± 0.07 *	8.16 ± 0.21	9.82 ± 0.25	36.65 ± 0.78	39.88 ± 1.01
8	6.89 ± 0.17	2.60 ± 0.07 **	1.26 ± 0.00	0.52 ± 0.01 *	85.47 ± 1.45	68.21 ± 0.58 **	1.22 ± 0.15	14.20 ± 0.72 **	11.69 ± 0.30	8.34 ± 0.11 **	-	-	6.32 ± 0.12	7.22 ± 0.11
9	10.13 ± 0.26	4.87 ± 0.12 **	13.84 ± 0.04	10.15 ± 0.03 *	98.30 ± 1.66	92.92 ± 0.79 *	1.06 ± 0.13	8.07 ± 0.41 **	6.31 ± 0.16	4.94 ± 0.08 *	2.93 ± 0.07	3.51 ± 0.09	17.71 ± 0.40	18.11 ± 0.46
10	9.06 ± 0.23	4.10 ± 0.10 **	4.91 ± 0.01	2.56 ± 0.01 **	73.79 ± 1.25	55.96 ± 0.47 **	5.99 ± 0.15	9.44 ± 0.48 **	4.61 ± 0.12	3.21 ± 0.04 *	4.52 ± 0.11	4.68 ± 0.12	41.02 ± 1.04	41.82 ± 1.06

* *p* < 0.05 and ** *p* < 0.01, compared to CAR group.

**Table 4:** Details for sampling of *Anemarrhenae rhizoma.*

No.	Place of Collection	Collection Time	Growing Condition
1	Changzhi, Shanxi	2014.09	Cultivated
2	Anguo, Hebei	2014.10	Cultivated
3	Bozhou, Anhui	2014.08	Cultivated
4	Huludao, Liaoning	2014.11	wild
5	Chifeng, Neimenggu	2014.12	wild
6	Changzhi, Shanxi	2015.03	Cultivated
7	Anguo, Hebei	2015.04	Cultivated
8	Datong, Shanxi	2015.03	Cultivated
9	Bozhou, Anhui	2015.04	Cultivated
10	Chifeng, Neimenggu	2015.05	wild
